# HES1 promotes aerobic glycolysis and cancer progression of colorectal cancer via IGF2BP2-mediated GLUT1 m6A modification

**DOI:** 10.1038/s41420-023-01707-4

**Published:** 2023-11-13

**Authors:** Jiayu Wang, Mengxin Zhu, Jinghan Zhu, Juntao Li, Xingchao Zhu, Kun Wang, Kanger Shen, Kexi Yang, Xiangyu Ni, Xin Liu, Guangbo Zhang, Qinhua Xi, Tongguo Shi, Weichang Chen

**Affiliations:** 1 Jiangsu Institute of Clinical Immunology, The First Affiliated Hospital of Soochow University, Suzhou, China; 2 Jiangsu Key Laboratory of Clinical Immunology, Soochow University, Suzhou, China; 3 Department of Gastroenterology, The First Affiliated Hospital of Soochow University, Suzhou, China

**Keywords:** Methylation, Cancer metabolism

## Abstract

Aerobic glycolysis has been shown to play a key role in tumor cell proliferation and metastasis. However, how it is directly regulated is largely unknown. Here, we found that HES1 expression was significantly higher in CRC tissues than that in adjacent normal tissues. Moreover, high HES1 expression is associated with poor survival in CRC patients. HES1 knockdown markedly inhibited cell growth and metastasis both in vitro and in vivo. Additionally, silencing of HES1 suppressed aerobic glycolysis of CRC cells. Mechanistic studies revealed that HES1 knockdown decreased the expression of GLUT1, a key gene of aerobic glycolysis, in CRC cells. GLUT1 overexpression abolished the effects of HES1 knockdown on cell aerobic glycolysis, proliferation, migration and invasion. ChIP-PCR and dual-luciferase reporter gene assay showed that HES1 directly bound the promoter of IGF2BP2 and promoted IGF2BP2 expression. Furthermore, our data indicated that IGF2BP2 recognized and bound the m^6^A site in the GLUT1 mRNA and enhanced its stability. Taken together, our findings suggest that HES1 has a significant promotion effect on CRC aerobic glycolysis and progression by enhancing the stability of m^6^A-modified GLUT1 mRNA in an IGF2BP2-dependent manner, which may become a viable therapeutic target for the treatment of CRC in humans.

**Figure:**
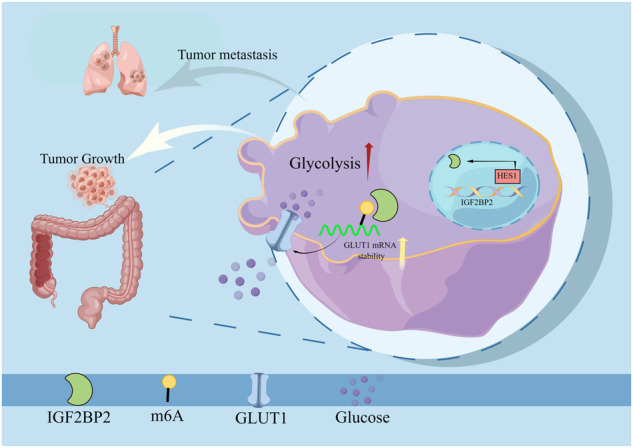
The mechanism of HES1 regulating glycolysis in CRC.

The mechanism of HES1 regulating glycolysis in CRC.

## Introduction

Colorectal cancer (CRC) is a common digestive tract malignant tumor that seriously threatens human life and health. According to cancer statistical analysis, there will be more than 59,000 new cases of CRC in China [1]. Late-stage diagnosis of CRC and high rates of metastasis are leading causes of death [2]. In spite of advances in therapies such as multidisciplinary therapy and targeted immunotherapy, five-year overall survival for patients with CRC remains low [3, 4]. Therefore, an in-depth study of the molecular mechanisms of CRC progression is expected to improve the prognosis of CRC patients.

Metabolic reprogramming is an important hallmark of cancer cells. Aerobic glycolysis is a phenomenon in which tumor cells prefer to meet their energy needs by consuming large amounts of glucose while producing lactate, even in normoxic environments [5, 6]. Enhanced glycolysis is mainly due to increased expression or activity of glycolysis-related genes in tumor cells [7, 8]. For example, glucose transporter 1(GLUT1), as the name suggests, transports glucose into the cytoplasm, initiating glycolysis. Research has shown that GLUT1 is highly expressed in various types of tumor tissues and is associated with poor clinical outcomes [9, 10]. Ample evidence indicated that abnormal glycolysis levels promote cancer cell proliferation and metastasis [11], but the molecular mechanisms modulating aerobic glycolysis have not been elucidated.

As a transcription factor, Hairy and enhancer of split homolog-1 (HES1) is one of the important members of the helix-loop-helix structural protein family, which plays a key role in cell differentiation, proliferation and other physiological processes [12]. Pathologically, HES1 is highly expressed in various cancers, such as pancreatic cancer, CRC, and non-small cell lung cancer, which are associated with poor outcomes [13–15]. Li et al. noted that in triple-negative breast cancer, HES1 modulated breast cancer stem cell (BCSC) self-renewal, BCSC population, and cancer cell proliferation through transcriptional activating Slug expression [16]. Furthermore, HES1 overexpression enhanced the chemoresistance of CRC to 5- fluorouracil by promoting epithelial-mesenchymal transition (EMT) and inducing the expression of ABC transporter genes including ABCC1, ABCC2 and P-gp1 [17]. These aforementioned findings showed that HES1 exerts a vital effect on tumor progression. However, the roles and underlying molecular mechanisms of HES1 in CRC remain ill-defined.

In this study, we analyzed the expression of HES1 in human CRC tissues and determined that HES1 plays an oncogenic role in CRC progression. Moreover, how HES1 controlled proliferation, metastasis and aerobic glycolysis in CRC cells and the mechanisms involved were explored. Our results demonstrated that HES1 was highly expressed in tumor tissues of CRC patients and upregulation of HES1 predicted a worse prognosis. Silencing of HES1 curbed the proliferation, metastasis and aerobic glycolysis of CRC cells. Importantly, we found that HES1 modulated the stability of GLUT1 mRNA by Insulin-like growth factor 2 binding protein 2 (IGF2BP2), an important reader of N6-methyladenosine (m^6^A) modification.

## Results

### HES1 expression is increased in CRC patients and is associated with poor survival in patients with CRC

**Fig. 1:**
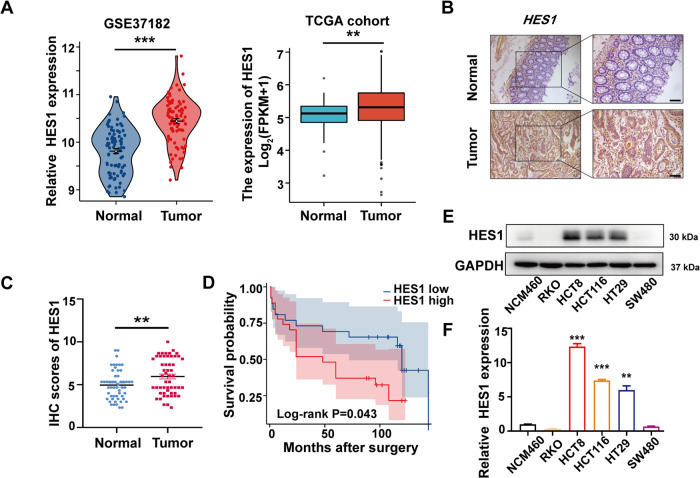
HES1 is highly expressed in CRC tissues and positively correlates with poor prognosis in CRC patients. **A** Expression of HES1 in CRC tissues (*n* = 84) and adjacent normal tissues (*n* = 88) based on a GSE37182 dataset. Expression of HES1 in CRC tissues (*n* = 647) and adjacent normal tissues (*n* = 51) based on a TCGA dataset. **B** Representative IHC image of HES1 in CRC tumor tissues and matched adjacent normal tissues (scale bar, 100 μm). **C** HES1 protein expression based on its staining index in normal adjacent tissues (*n* = 53) and CRC samples (*n* = 53). **D** Relationship between HES1 expression and survival prognosis in CRC patients. The protein (**E**) and mRNA (**F**) levels of HES1 in different CRC cell lines and normal colon epithelial cells. The data represent the mean ± SEM. **P < 0.01, ***P < 0.001.

We investigated the mRNA expression of HES1 in the tissue samples of CRC patients using GEO and TCGA datasets. The results showed that HES1 was highly expressed in CRC tumor tissues (Fig. 1A). Subsequently, we examined the protein expression of HES1 in CRC patient specimens in our cohort by IHC assay. As shown in Fig. 1B, C, compared with the corresponding normal adjacent tissues, the protein levels of HES1 were significantly increased in the tumor tissues. Furthermore, the relationships between HES1 expression and clinicopathological characteristics in CRC patients were examined. The HES1 levels in advanced (III and IV stages) CRC patients were much higher than those in early stages (I and II) patients (Table 1). In addition, CRC patients with lymph node metastasis had higher HES1 levels compared with those without lymph node metastasis (Table 1). Importantly, CRC patients with high HES1 expression had a worse prognosis (Fig. 1D). Moreover, we observed that the HES1 expression in most CRC cell lines (HCT8, HCT116, and HT29) was markedly higher than that in the colonic epithelial cell line NCM-460 (Fig. 1E, F). Based on these results, the expression of HES1 is increased in both CRC patients and CRC cell lines, and increased HES1 expression is associated with the progression of CRC.

**Table 1:** Correlation between HES1 expression and clinical features in patients with CRC (*n* = 53).

Characteristic Total NO.		HES1 expression		*P* value
		High	Low	
**All case**	53	27	26	
Age				0.6586
>60	34	15	16	
≤60	19	12	10	
Gender				0.2883
Male	35	16	19	
Female	18	11	7	
Tumor depth				0.6687
T1/T2	5	2	3	
T3/T4	48	25	23	
LN involvement				0.0176*
N0	30	11	19	
N1/N2	23	16	7	
Metastasis				0.4906
M0	51	25	26	
M1	2	2	0	
TNM stage				0.0084*
I/II	29	10	19	
III/IV	24	17	7	

### HES1 knockdown inhibits the malignant phenotype of CRC

**Fig. 2:**
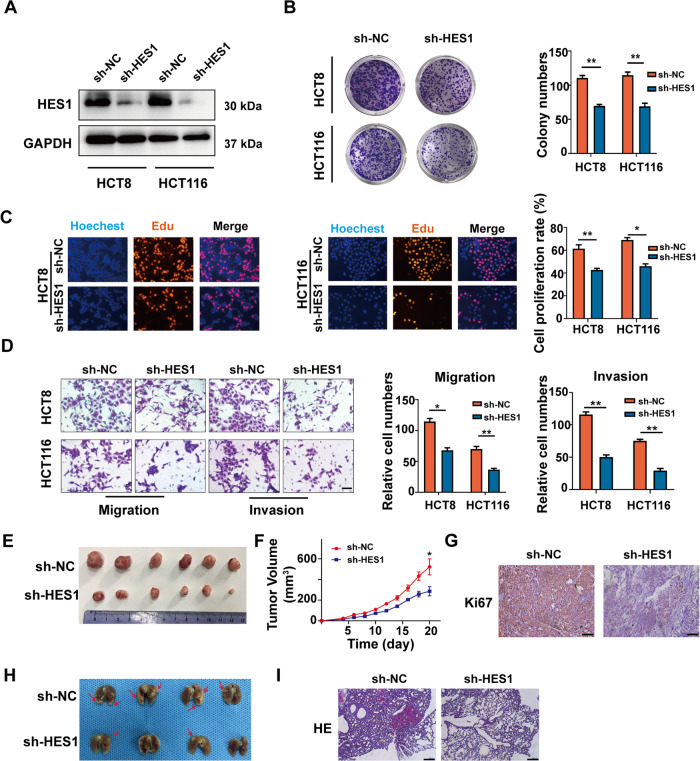
Knockdown of HES1 inhibits the malignant behavior of CRC cells invitro and in vivo. **A** Western blot analysis of HES1 in CRC stable cell lines with HES1 inhibition (shB7-H3) or their control cell lines (sh-NC). GAPDH was used as a load control. Colony formation (**B**) and EdU (**C**) assay of HES1 knockdown HCT8 and HCT116 cells. **D** Cell migration and invasion in HES1 knockdown HCT8 and HCT116 cells were examined by transwell assays. **E** Representative images of subcutaneous xenografts from the sh-NC (*n* = 6) and sh-HES1 groups (*n* = 6). **F** The growth curves of tumors formed by the sh-NC and sh-HES1 cells. **G** One representative image of the IHC analysis of KI67 in the tissues of subcutaneous xenografts from the sh-NC (*n* = 6) and sh-HES1 groups. **H** The effects of HES1 knockdown on the metastasis of HCT116 cells in vivo. Lungs were observed for metastatic nodules on the surface, stained by H&E for histological analyses, arrows point to metastatic nodules. Representative photographs and H&E staining (**I**) were shown (*n* = 4 mice per group). Scale bar, 100 μm. The data represent the mean ± SD. *P < 0.05, **P < 0.01, ***P < 0.001.

Next, we tested changing the HES1 expression pairs malignant behavior of CRC cells. Three non-overlapping siRNAs targeting HES1 (HES1 siRNA-1, HES1 siRNA-2 and HES1 siRNA-3) were used to silence the HES1 expression in HCT8 and HCT116 cells. Among these siRNAs, HES1 siRNA-1 had the greatest inhibitory effect on HES1 expression in CRC cells and was chosen to construct the shRNA lentivirus (sh-HES1, Supplementary Fig. S1). As shown in Fig. 2A, sh-HES1 mediated stably reducing HES1 expression in HCT8 and HCT116 cells. When the HES1 expression was knocked down, cell proliferation was significantly decreased in HCT8 and HCT116 cells (Fig. 2B, C), and the migration and invasion were also decreased (Fig. 2D).

To further determine the role of HES1 in CRC, control cells (sh-NC) and HES1 silencing HCT116 cells (sh-HES1) were injected subcutaneously into nude mice. HES1 knockdown significantly inhibited HCT116 tumor growth (Fig. 2E, F). IHC staining showed that the Ki-67 levels were significantly decreased in the xenograft tissues of the HES1 knockdown group (Fig. 2G). Additionally, to explore the effect of HES1 on CRC metastasis in vivo, a caudal vein injection model was established. As shown in Fig. 2H, the mice with HES1 silencing HCT116 cells, but not control cells, formed fewer metastatic nodules in the lungs. The presence of lung metastases from CRC was confirmed by HE staining (Fig. 2I).

### HES1 modulates aerobic glycolysis in CRC cells

**Fig. 3:**
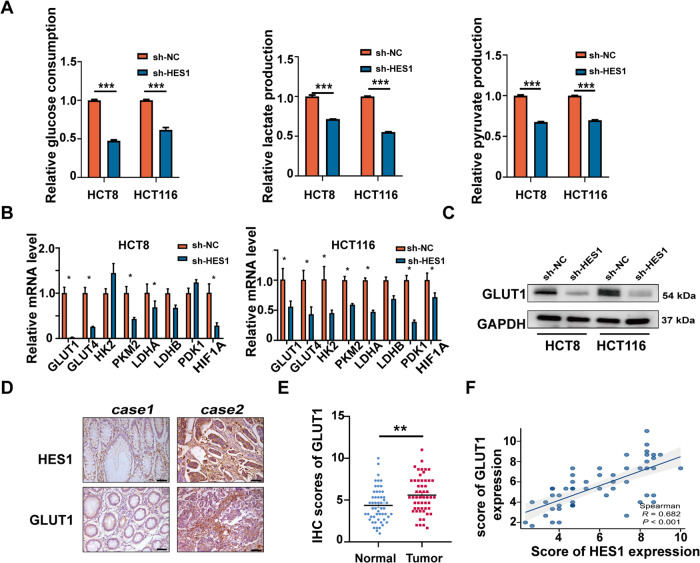
HES1 knockdown inhibits aerobic glycolysis and GLUT1 expression in CRC. **A** Glucose consumption, lactate production, and pyruvate production were measured in sh-HES1 HCT8 and HCT116 cells. **B** The mRNA expression of glycolysis-related genes in sh-HES1 HCT8 and HCT116 cells was detected by RT-qPCR. **C** The GLUT1 protein level in sh-HES1 HCT8 and HCT116 cells was detected by western blot. **D** IHC analysis of HES1 and GLUT1 protein expression in tissue samples of CRC patients. Representative images are shown. **E** HES1 protein expression based on its staining index in normal adjacent tissues (*n* = 53) and CRC samples (*n* = 53). **F** Correlation analysis of HES1 and GLUT1 protein expression in human CRC samples.The data represent the mean ± SD. *P < 0.05, **P < 0.01, ***P < 0.001.

Given that aerobic glycolysis plays an important role in the progression of CRC, we investigated whether HES1 is involved in the glycolysis process. As shown in Fig. 3A, knockdown of HES1 inhibited glucose uptake, lactate and pyruvate production in HCT8 and HCT116 cells. The results of qRT-PCR showed that HES1 depletion led to a marked decrease in a series of key glycolysis-related genes (GLUT1, GLUT4, HK2, PKM2, LDHA, LDHB, PDK1 and HIF-1α) in HCT8 and HCT116 cells (Fig. 3B). Furthermore, western blot analysis showed that knockdown of HES1 significantly reduced the protein level of GLUT1 in HCT8 and HCT116 cells (Fig. 3C). We then analyzed the correlation between HES1 and GLUT1 levels in CRC clinical specimens. Compared with the corresponding normal adjacent tissues, GLUT1 was highly expressed in tumor tissues (Fig. 3D, E). Importantly, the expression of GLUT1 was positively correlated with HES1 expression in CRC tissue samples (Fig. 3F). Overall, HES1 controls aerobic glycolysis in CRC.

### HES1 regulates glycolysis, growth, and metastasis of CRC cells through GLUT1

**Fig. 4:**
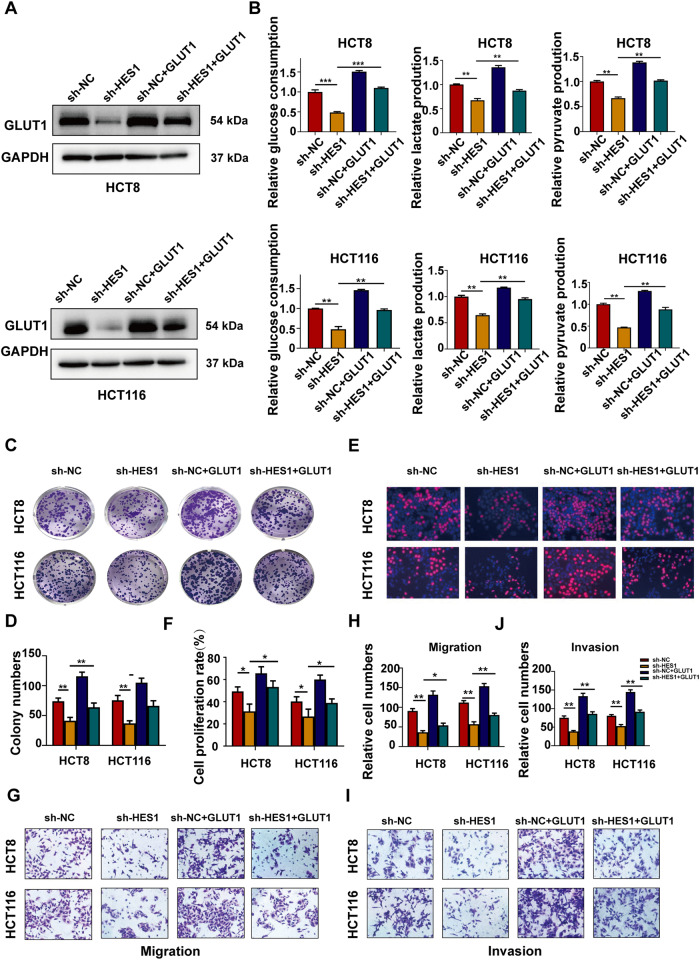
GLUT1 participates in HES1-mediated aerobic glycolysis and malignant behavior of CRC cells. **A** Western blot analysis of GLUT1 protein levels in sh-HES1 HCT8 and HCT116 cells after transfection with negative control (NC) or GLUT1 overexpression plasmids. GAPDH was used as a negative control. **B** Glucose consumption, lactate production and pyruvate production were measured in sh-HES1 HCT8 and HCT116 cells after transfection with NC or GLUT1 overexpression plasmids. **C**, **E** Colony formation (**C**, **D**) and EdU (**E**, **F**) assay of sh-HES1 HCT8 and HCT116 cells after transfection with NC or GLUT1 overexpression plasmids. **G**, **I**. The migration (**G**, **H**) and invasion (**I**, **J**) ability of sh-HES1 HCT8 and HCT116 cells after transfection with NC or GLUT1 overexpression plasmids. The data represent the mean ± SD. *P < 0.05, **P < 0.01, ***P < 0.001.

To explore whether HES1 regulated glycolysis, growth, and metastasis of CRC through GLUT1, a commercial GLUT1 overexpression plasmid was used to transfect into HES1 knockdown CRC cells. The results of western blot confirmed that transfection with GLUT1 overexpression plasmids significantly increased the GLUT1 protein expression in HES1 knockdown HCT8 and HCT116 cells (Fig. 4A). As shown in Fig. 4B, GLUT1 overexpression reversed the inhibition of HES1 knockdown on glucose uptake, lactate and pyruvate production in HCT8 and HCT116 cells. Moreover, overexpression of GLUT1 in both cell lines significantly rescued the inhibitory effects of HES1 knockdown on cell growth and metastasis (Fig. 4C–J).

### HES1 modulates GLUT1 expression via IGF2BP2

**Fig. 5:**
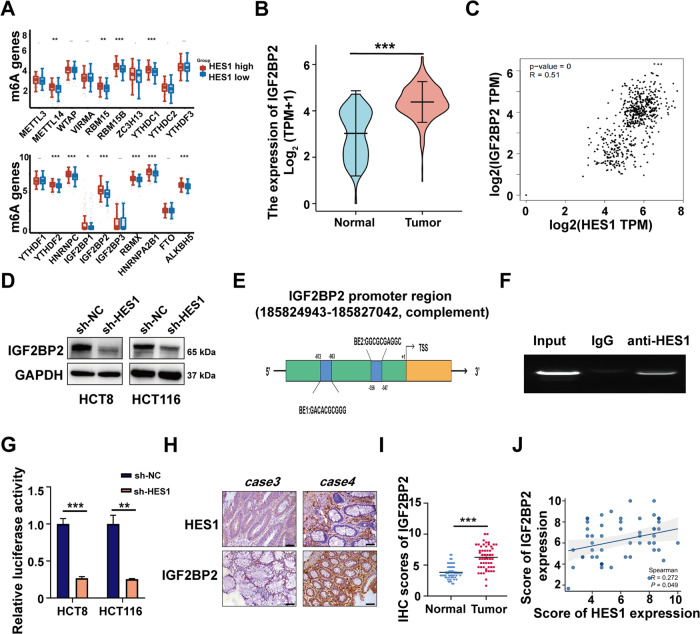
HES1 directly promotes IGF2BP2 transcription. **A** The mRNA levels of 20 m^6^A-related genes were analyzed in HES1 low and HES1 high group based on a TCGA database. Red and blue indicate relatively high and low HES1 expression. **B** Expression of IGF2BP2 in CRC tissues (*n* = 647) and adjacent normal tissues (*n* = 51) based on a TCGA dataset. **C** Correlation between IGF2BP2 and HES1 expression in CRC tissue samples based on a TCGA dataset. **D** The protein expression of IGF2BP2 in sh-HES1 HCT8 and HCT116 cells was detected by western blot. GAPDH was used as a negative control. **E** The JASPAR website predicts the HES1 binding sites in the IGF2BP2 promoter region. There are two HES1 binding sites in the 5’ region of IGF2BP2: BE1 (−972 nt to − 963 nt), BE2 (−356 nt to −347 nt). **F** ChIP analysis of HES1 binding to IGF2BP2 promoter in HCT8 cells. Normal mouse IgG was used as a control. **G** Luciferase reporter gene was used to determine the luciferase activity of IGF2BP2 promoter in knockdown HCT8 and HCT116 cells. **H** IHC analysis of HES1 and IGF2BP2 protein expression in CRC patient samples. Representative images are shown. **I** IGF2BP2 protein expression based on its staining index in normal adjacent tissues (*n* = 53) and CRC samples (*n* = 53). **J** Correlation analysis of HES1 and IGF2BP2 protein expression in human CRC samples. The data represent the mean ± SD. *P < 0.05, **P < 0.01, ***P < 0.001.

**Fig. 6:**
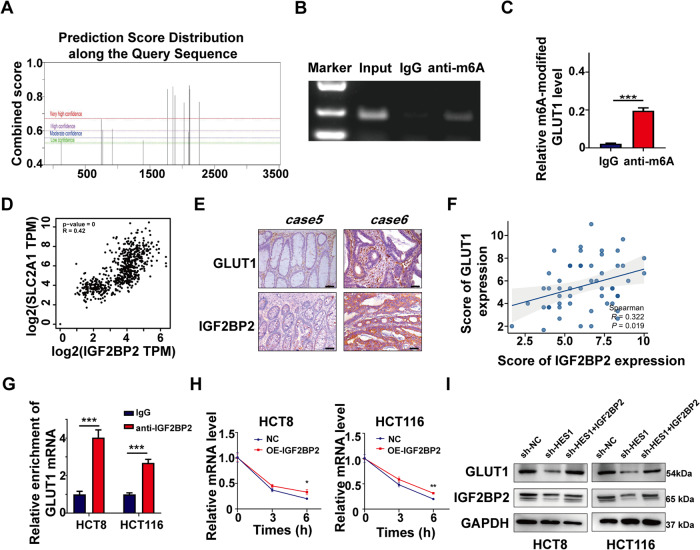
IGF2BP2 promotes GLUT1 expression in an m6A-dependent manner. **A** The SRAMP website predicts the presence of m6A modification sites on GLUT1 mRNA. **B**, **C** MeRIP-PCR analysis of anti-m^6^A antibody enriching GLUT1 mRNA. **D** Correlation between IGF2BP2 and GLUT1 in CRC based on a TCGA database. **E** IHC analysis of GLUT1 and IGF2BP2 protein expression in CRC patient samples. Representative images are shown. **F** Correlation analysis of IGF2BP2 and GLUT1 protein expression levels in human CRC samples. **G** RIP-qPCR analysis of IGF2BP2 binding to the GLUT1 mRNA. IgG was used as a negative control. **H** GLUT1 mRNA expression was analyzed after transfection of IGF2BP2 in HCT8 and HCT116 cells at a predetermined time after actinomycin D (5 μg/ml) treatment. **I** Western blot analysis of GLUT1 protein in sh-HES1 HCT8 and HCT116 cells transfected with IGF2BP2 overexpression plasmids. The data represent the mean ± SD. *P < 0.05, **P < 0.01, ***P < 0.001.

To investigate the mechanism of HES1-mediated expression of GLUT1 in CRC cells, we reviewed works of literature. IGF2BP2, as an N6- methyladenosine (m^6^A) reader, has been reported to be able to directly bind to and stabilize GLUT1 mRNA in pancreatic ductal adenocarcinoma and CRC [18]. Based on the TCGA database, we found that compared with the HES1 low expression group, 11 out of 20 m6A regulatory genes were significantly up-regulated in CRC tissues, with IGF2BP2 having the most significant difference (Fig. 5A). Hence, we hypothesized that HES1-induced GLUT1 expression may be associated with IGF2BP2. Moreover, TCGA data analysis showed that IGF2BP2 was highly expressed in CRC and showed a significant positive correlation with HES1 (Fig. 5B, C). In addition, the expression of IGF2BP2 was significantly decreased in HES1 knockdown HCT8 and HCT116 cells (Fig. 5D). Then, we used the JASPAR website and found that there were 2 HES1 binding sites in the IGF2BP2 gene promoter region (Fig. 5E). Furthermore, the ChIP-PCR results revealed that compared with the IgG group, anti-HES1 could enrich more IGF2BP2 (Fig. 5F). And dual-luciferase reporter gene assay showed that the knockdown of HES1 could remarkedly reduce the luciferase activity of the IGF2BP2 gene promoter in HCT8 and HCT116 cells (Fig. 5G). Thus, these results suggest that knockdown of HES1 inhibits IGF2BP2 transcription. Next, the correlation between HES1 and IGF2BP2 levels in CRC clinical specimens was analyzed. IGF2BP2 is highly expressed in tumor tissues (Fig. 5H, I). An important finding was that HES1 expression was positively correlated with the expression of IGF2BP2 in CRC tissues (Fig. 5J). We further confirmed the role of IGF2BP2 in regulating GLUT1 in CRC cells. The SRAMP website was used to predict the m6A site on GLUT1 mRNA (Fig. 6A). Then, we verified that anti-m6A antibody significantly enriched GLUT1 mRNA in HCT116 cells by MeRIP-PCR method (Fig. 6B, C). TCGA data analysis showed that there was a significant positive correlation between IGFBP2 expression and GLUT1 expression (Fig. 6D). In addition, we observed a positive correlation between IGF2BP2 and GLUT1 expression in CRC tissue specimens in our cohort (Fig. 6E, F). The results of RIP assay showed that compared with the IgG group, more GLUT1 mRNA was enriched in the anti-IGF2BP2 group (Fig. 6G). Moreover, overexpression of IGF2BP2 could enhance the stability of GLUT1 mRNA (Fig. 6H). Importantly, we observed that overexpression of IGF2BP2 ultimately abolished the inhibition of HES1 deleption on GLUT1 protein expression in HCT8 and HCT116 cells (Fig. 6I). These data indicates that HES1 regulates GLUT1 expression in CRC cells in an m^6^A-IGF2BP2 dependent manner.

## Discussion

HES1 has been reported to be overexpressed in multiple somatic tumors and its dysregulated expression led to the occurrence and development of cancer [19, 20]. However, the expression and roles of HES1 in CRC remain to be illustrated. High expression of HES1 was significantly correlated with distal metastasis at diagnosis and its high expression is a poor prognostic factor for CRC patients [21]. Weng et al. noted that high Hes1 mRNA expression was associated with poor prognosis in CRC patients [22]. Consistent with these findings, we found that both mRNA and protein expression of HES1 was highly expressed in the CRC and correlated with TNM staging in CRC patients. Importantly, in our cohort of CRC patients, patients with high HES1 expression showed significantly poor prognosis. However, there are controversial research results on the clinicopathological associations and prognostic significance of HES1 in CRC. The study by Gilhyang Kim et al. showed that low HES1 expression was obviously correlated with large tumor size, lymphovascular invasion, distant metastasis, and an unfavorable prognosis [23]. Additionally, a previous study noted that loss of nuclear expression of HES1 in colorectal adenocarcinoma was markedly associated with female sex, right-sided location, BRAFV600E mutation, microsatellite instability, larger tumor size, and worse survival [24]. The inconsistent data on the prognostic value of HES1 in CRC patients might be associated with the CRC subtypes and intracellular location. The real prognostic value of HES1 in CRC warrants further confirmation.

Several studies have demonstrated that HES1 is involved in the regulation of various biological processes in cancers, such as cell stem maintenance, proliferation, and metastasis [12]. Gao et al. have demonstrated that HES1 overexpression upregulated the expression of stemness-related genes in colon cancer cells, elevated the self-renewal properties of the stem-like cells, and enhanced the ability of tumor sphere formation [13]. In addition, knocking down HES1 induced CRC cell senescence and decreased the invasion ability through the STAT3-MMP14 pathway [22]. Herein, we observed that HES1 silencing remarkedly suppressed cell proliferation, migration and invasion of CRC cells in vitro. Moreover, HES1 knockdown HCT116 cells showed the decreased ability of xenograft growth and lung metastasis in vivo.

Although a number of studies have demonstrated that HES1 functioned as an oncogene in CRC by promoting tumor proliferation and metastasis, the molecular mechanisms underlying the pro-tumor effects of HES1 are largely unknown. Aerobic glycolysis has been shown to play a crucial role in tumor cell proliferation and metastasis in cancers [25, 26]. Chen et al. showed that LncRNA KCNQ1OT1 promoted the occurrence of CRC by promoting aerobic glycolysis through HK2 [27]. In addition, FSTL3-mediated β-catenin pathway activation promoted CRC aerobic glycolysis, thereby affecting tumor invasion and metastasis ability [28]. Notch-HES1 axis can improve the glycolysis rate, increase the proliferation efficiency and maintain the pluripotency of human adipose tissue-derived multiline progenitor cells. In the present study, we found that knocking down HES1 inhibited glucose uptake, lactate production and major genes including GLUT1 involved in glycolysis in CRC cells, suggesting that HES1 plays important roles in modulating aerobic glycolysis in CRC. Overexpression of GLUT1 significantly alleviated the inhibitory effect of HES1 knockout on cell growth and metastasis. Moreover, GLUT1 is highly expressed in CRC and positively correlated with HES1 in CRC tissue samples. These results suggested that HES1 could contribute to cell growth and metastasis of CRC cells by regulating aerobic glycolysis through GLUT1.

As one of the epigenetic regulatory mechanisms, m^6^A modification is an emerging research frontier in tumor biology and plays critical roles in regulating mRNA fate [29, 30]. Three types of proteins (‘writers’, ‘readers’ and erasers) participated in the m^6^A modification processes [31]. It is well-known that IGF2BP2 is the m^6^A ‘readers’ that recognizes m^6^A-modified mRNAs and modulates RNA localization, translation, and stability [32–34]. Xu et al. noted that IGF2BP2 served as a reader for m^6^A-modified lncRNA DANCR and stabilized DANCR RNA, promoting cancer stemness-like properties and pancreatic cancer pathogenesis [31]. In head and neck squamous carcinoma (HNSCC), IGF2BP2 recognized and bound the m^6^A site in the coding sequence region of Slug and elevated its mRNA stability, which promotes lymphatic metastasis and epithelial-mesenchymal transition of HNSCC cells [35]. Given that IGF2BP2 could directly bind to and stabilize GLUT1 mRNA in pancreatic ductal adenocarcinoma and CRC [18], we inferred whether HES1 modulated GLUT1 expression via IGF2BP2 in an m^6^A modification-dependent manner. We observed that the levels of HES1 were positively related to the IGF2BP2 expression in tissue samples of patients with CRC. The ChIP-PCR and dual-luciferase reporter gene assay demonstrated that HES1 directly bound the promoter of IGF2BP2. Moreover, HES1 deleption led to a significant decrease in IGF2BP2 expression in CRC cells. These data suggest that HES1 is an essential transcriptional activator of IGF2BP2 in CRC. To investigate the relation between IGF2BP2 and GLUT1 in CRC, MeRIP-PCR, RIP-qPCR and RNA stability experiments were performed. The results indicated that IGF2BP2 could directly bind the m6A site in the GLUT1 mRNA and enhance its stability. Furthermore, IGF2BP2 overexpression reversed the effects of HES1 knockdown on GLUT1 expression. Therefore, these data suggest that HES1 positively modulated GLUT1 expression in CRC cells through IGF2BP2 recognizing and binding the m6A site in the GLUT1 mRNA and enhancing its stability. However, further molecular mechanisms underlying HES1 modulating GLUT1 deserve extensive study.

In summary, this study confirmed that HES1 was overexpressed in CRC patients and was associated with malignant behavior. Moreover, HES1 knockdown suppressed proliferation, metastasis, and aerobic glycolysis of CRC cells via reducing the stability of m^6^A-modified GLUT1 mRNA in an IGF2BP2-dependent manner. Hence, the therapeutic intervention of the HES1/IGF2BP2/GLUT1 axis-mediated aerobic glycolysis might be a novel strategy for arresting uncontrolled growth and metastasis in CRC.

## Materials and methods

### Patient samples

Paraffin specimens of 53 CRC tissues and their adjacent non-tumor tissues were collected from the First Affiliated Hospital of Soochow University. The study was approved by the Ethics Committee of Soochow University (reference number: 2021-327), and informed consent was obtained from each patient. Detailed clinicopathological information of CRC patients is provided in Supplementary Table S1.

### Microarray data

Microarray gene expression data set GSE37182 from GEO database (https://www.ncbi.nlm.nih.gov/geo/), download the data format for MINiML. From the TCGA database (https://portal.gdc.cancer.gov) to download and organize CRC RNAseq data and extract the TPM format of the data. Expression analysis was performed using R software (package: ggplot2).

### Immunohistochemistry (IHC)

IHC staining was performed as described previously [36]. Simply put, the paraffin-embedded tissue was cut into 5 μm-thick sections, followed by dewaxing, high-temperature antigen retrieval, non-specific antigen blocking, overnight incubation with anti-HES1 (CST, Danvers, MA, USA), anti-GLUT1(Proteintech, Wuhan, China), anti-IGF2BP2(Proteintech) or anti-Ki67 (Proteintech) at 4 °C. Finally, the sections were incubated with HRP-conjugated secondary antibody and developed with diaminobenzidine (DAB) solution. These slides are photographed under a microscope. Staining intensity (0, negative; 1, weak; 2, medium; 3, strong) and staining ratio (1, <25%; 2, 25–50%; 3, 50–75%; 4, >75%) were evaluated.

### Western blotting

Total protein was isolated from cells using cell lysate (Beyotime) containing protease and phosphatase inhibitors. The protein concentration was measured using the BCA protein assay kit (Beyotime). Primary antibodies specifc to HES1 (1:1000), GLUT1 (1:1000), IGF2BP2(1:1000), GAPDH (Abclonal, Wuhan 1:5000) were used. Last, we detected the signal by using an Enhanced Chemiluminescence (ECL) system according to the manufacturer’s instructions.

### Cell lines and cell cultures

The human normal colonic epithelial cell line NCM-460 and human CRC cell lines (HCT116, HT29, RKO, HCT8, and SW480) were purchased from American Type Culture Collection (ATCC, USA). All cell lines were cultured in DMEM (Eallbio, Beijing, China) containing 10% fetal bovine serum (FBS, Eallbio) and 1% penicillin-streptomycin (Beyotime) in a humidified incubator with 5% CO_2_ at 37 °C.

### Cell transfection and infection

CRC cell lines were cultured in 6-well plates and transfected with HES1 specific siRNA (siRNA-1, −2, and −3) and siRNA-NC using lipofectamine 2000 (Invitrogen, USA) following the manufacturer’s instructions. Lentiviruses containing short hairpin RNA (shRNA) with HES1 siRNA-1 sequence came from GenePharma. Infection occurs when the colorectal cancer cells have grown to 30%. We verified the effectiveness by western blot 72 h later.

### Colony formation

CRC cells were seeded into 12-well plates at a density of 800 cells per well. After 2 weeks, colonies were fixed with 4% paraformaldehyde and stained with crystal violet.

### 5-Ethynyl-2′-deoxyuridine (EdU) incorporation assay

For EdU assay, cells in the logarithmic growth phase were seeded in 24-well plates and incubated with EdU working reagent (Beyotime) at the concentration of 10 μM for 2 h. Cells were washed twice with PBS for 5 min each and then incubated with 4% paraformaldehyde for 15 min. After washing twice with PBS for 5 min, the samples were permeabilized with 0.3% TritonX-100 and subsequently stained with the reaction solution. Hoechst33342 (Beyotime) was used for the nuclei staining. Finally, we counted the cells and photographed them under a fluorescence microscope.

### Transwell migration and invasion assays

During the migration experiment, cells (3×10^4^ cells) were re-suspended in a medium without fetal bovine serum and added to the upper chamber (8 μm pore size, BD Biosciences, NJ, USA). Then we added 500 μl cell medium containing 20% fetal bovine serum into lower chambers. To conduct the invasion experiment, diluted Matrigel (Corning) was first coated in the upper cavity. After incubation for 24 h, cells were fixed with paraformaldehyde 4% for 20 min and then stained with crystal violet (Beyotime) for 20 min. Finally, we counted the cells on the lower surface of the chamber and photographed them under an inverted microscope.

### Animal experiments

All the female BALB/c nude mice aged 6 to 8 weeks were obtained from the Laboratory Animal Center of Soochow University (Suzhou, China). Mice were acclimated to the new environment for one week before the experiment. The mice were randomly divided into two groups as sh-HES1 group (*n* = 6) and sh-NC group (*n* = 6). Sh-HES1 HCT116 cells (5×10^6^) and corresponding control cells were resuspended with 100 µl PBS and injected into the right subcutaneous BALB/c nude mice. Tumor size was measured every 3 days. The tumor volume was calculated by the formula V = 0.5 × a × b^2^, where V was equal to the tumor volume, while a and b represented the longitudinal and transverse diameter, respectively. Twenty days later, the mice were sacrificed to resect the tumors, then the tumors were weighed. Moreover, the tumors were used for IHC assay.

To establish a metastasis model, 6-week-old female BALB/c nude mice were given sh-HES1 HCT116 cells or control HCT116 cells (2 × 10^6^ cells per mouse) by caudal vein injection. 45 days after injection, mice were sacrificed and their lungs were isolated. Furthermore, the paraffin-embedded lungs were serially sectioned and after hematoxylin-eosin (HE) staining, they were observed through a microscope.

### Glucose uptake, lactate production and pyruvate production assay

The glucose, lactate, and pyruvate levels were measured using a glucose assay kit (Robio, Shanghai), a lactate assay kit (Jian cheng, Nanjing), and a pyruvate assay kit (Jian cheng) following the manufacturer’s protocols. Simply, the cells were inoculated into a 6-well plate and cultured for 24 h, and then the corresponding supernatant was collected for detection.

### Quantitative RT-PCR (qRT-PCR)

RNA extraction was performed using TRIZOL reagent (Vazyme) according to the manufacturer’s instructions. qRT-PCR detection of target genes using the SYBGreen method (Bio-Rad detector). The relative changes in transcript numbers in each sample were determined by normalizing mRNA levels by GAPDH. Gene-specific primer sequences are shown in Supplementary Table S2.

### MeRIP assays

MeRIP assay was performed using m6A RNA enrichment kit (Epigentek, USA). Briefly, the target fragment containing m6A was pulled down using beaded m6A capture antibody, and the enriched RNA was then released, purified and eluted. Finally, qRT-PCR was performed to quantify the methylation changes of target genes.

### RNA binding protein immunoprecipitation (RIP)

The RIP assay was performed using the RIP kit (BersinBio, Guangzhou, China) according to the manufacturer’s protocol. Simply put, the cells are cleaved on ice with a RIP lysis buffer. Whole-cell lysates were then incubated overnight with magnetic protein A/ G beads and 5 µg normal IgG antibody or IGF2BP2 antibody at 4 °C. Next, the RNA-protein complex was continuously rinsed with eluent and treated with protease K at 55 °C for 1 h. The binding RNA was extracted for qRT-PCR analysis. The primers were listed in Supplementary information, Table S1.

### Luciferase reporter assay

To measure the HES1 transcriptional activation, the luciferase reporter plasmids containing the promoter sequence (MiaoLing Plasmid Platform, Wuhan, China) were transfected in sh-NC and sh-HES1 cells. After 24 h, a Dual-Luciferase Reporter Assay System was used to measure the firefly and renilla luciferase activities. The relative luciferase activity was calculated by dividing Fluc by Rluc and was normalized to sh-NC.

### Chromatin Immunoprecipitation (ChIP)

ChIP was performed as described previously [37]. Briefly, cells were crosslinked with 1% formaldehyde and then cleaved with a cracking solution. The genomic DNA was processed by ultrasound to get a length of about 300–1000 bp. After the DNA fragments were incubated with anti-HES1 antibody or control IgG overnight at 4 °C, protein G Agarose was added to collect the antibody/antigen /DNA complex. Then, the complexes were undergone a series of buffer elution, and purified short hairpin DNA was detected by qRT-PCR and DNA agarose gel electrophoresis. The primer sequences for the IGF2BP2 promoter region were listed as below: Forward, 5’-CCCTCGGCACTTCCCAGAATAG-3’ Reverse, 5’-GCAAGGTGGACCGGATGTGA-3’.

### RNA stability assay

HCT8 or HCT116 cells were transfected with IGF2BP2 plasmid (MiaoLing Plasmid Platform) and then treated with actinomycin D at a final concentration of 5 μg/ml for 3 and 6 h. Total RNA was extracted and analyzed by qRT-PCR.

### Statistical analyses

In this study, all statistical data were analyzed using GraphPad Prism 8 (La Jolla, CA, USA). Data were expressed as mean ± SD or SEM. Differences between groups were determined by student t-test or one-way analysis of variance. *P* value of <0.05 was considered statistically significant.

### Supplementary information

supplementary information

Supplementary Figure S1

Original Data File

## Data Availability

The authors declare that all data generated or analyzed during this study are included in this published article. The data presented in this study are available on request from the corresponding authors.
