# Supplementary material for: HES1 promotes aerobic glycolysis and cancer progression of colorectal cancer via IGF2BP2-mediated GLUT1 m6A modification
Source: Cell Death Discov. 2023 Nov 13;9:411. doi: 10.1038/s41420-023-01707-4 (PMC10643658; doi:10.1038/s41420-023-01707-4)
Supplement: Supplementary file 1 — supplementary information [file 41420_2023_1707_MOESM1_ESM.docx]

**Supplementary Figure legends:**

**Supplementary Figure S1: Treatment with HES1 siRNA reduced the HES1 expression in CRC cells.** (A) The protein expression of HES1 in HCT8 and HCT116 cells after transfection with HES1 siRNAs (si-1, si-2 and si-3). (A) The mRNA expression of HES1 in HCT8 and HCT116 cells after transfection with HES1 siRNAs (si-1, si-2 and si-3). The data represent the mean ±SD. *P<0.05, **P<0.01, ***P<0.001.

**Supplementary Table S1：Primer sequences of RT-qPCR.**

| **Gene Name** | **Primer Sequence (5’-3’)** |
| --- | --- |
| GAPDH | F: GGAGCGAGATCCCTCCAAAAT |
|  | R: GGCTGTTGTCATACTTCTCATGG |
| LDHA | F: ATGGCAACTCTAAAGGATCAGC |
|  | R: CCAACCCCAACAACTGTAATCT |
| LDHB | F: TGGTATGGCGTGTGCTATCAG |
|  | R: TTGGCGGTCACAGAATAATCTTT |
| GLUT1 | F: GGCCAAGAGTGTGCTAAAGAA |
|  | R: ACAGCGTTGATGCCAGACAG |
| HK2 | F: GAGCCACCACTCACCCTACT |
|  | R: CCAGGCATTCGGCAATGTG |
| PKM2 | F: ATGTCGAAGCCCCATAGTGAA |
|  | R: TGGGTGGTGAATCAATGTCCA |
| PDK1 | F: CTGTGATACGGATCAGAAACCG |
|  | R: TCCACCAAACAATAAAGAGTGCT |
| GLUT4 | F: TGGGCGGCATGATTTCCTC |
|  | R: GCCAGGACATTGTTGACCAG |
| HIF1A | F:GAACGTCGAAAAGAAAAGTCTCG |
|  | R:CCTTATCAAGATGCGAACTCACA |
| HES1 | F:TCAACACGACACCGGATAAAC |
|  | R:GCCGCGAGCTATCTTTCTTCA |
