# Supplementary figures and images for: HES1 promotes aerobic glycolysis and cancer progression of colorectal cancer via IGF2BP2-mediated GLUT1 m6A modification
Source: Cell Death Discov. 2023 Nov 13;9:411. doi: 10.1038/s41420-023-01707-4 (PMC10643658; doi:10.1038/s41420-023-01707-4)

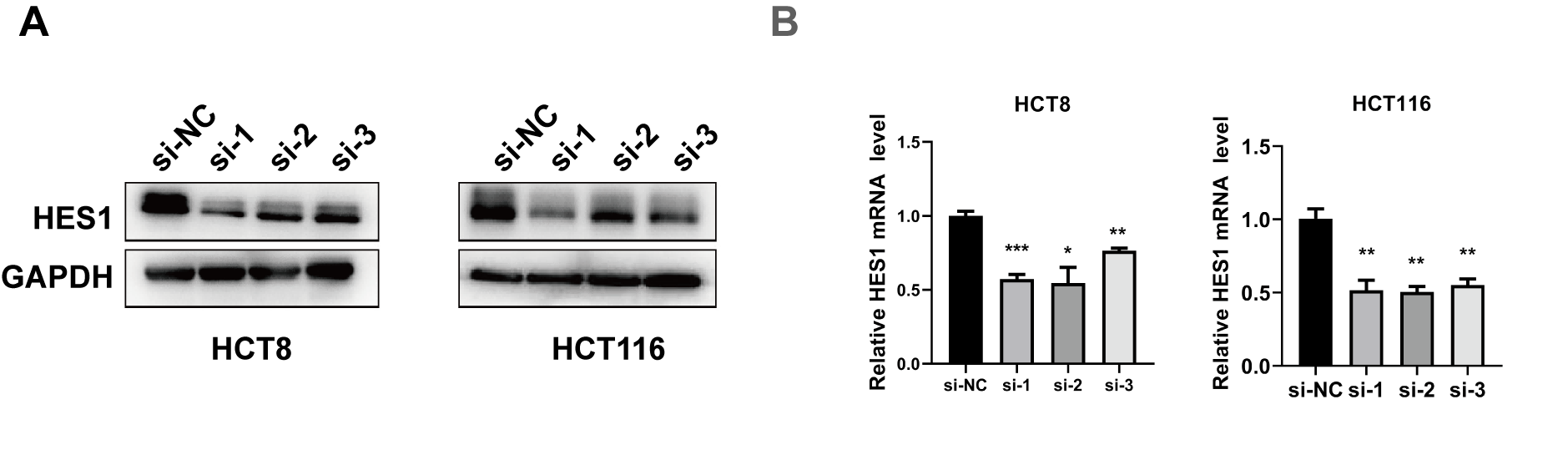

Supplement: Supplementary file 2 — Supplementary Figure S1 [file 41420_2023_1707_MOESM2_ESM.png]

Figure1E:


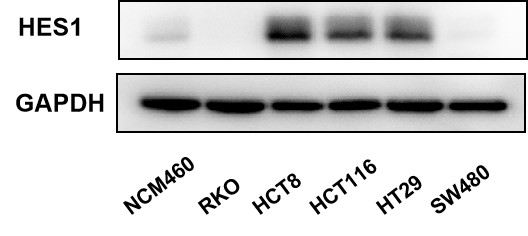


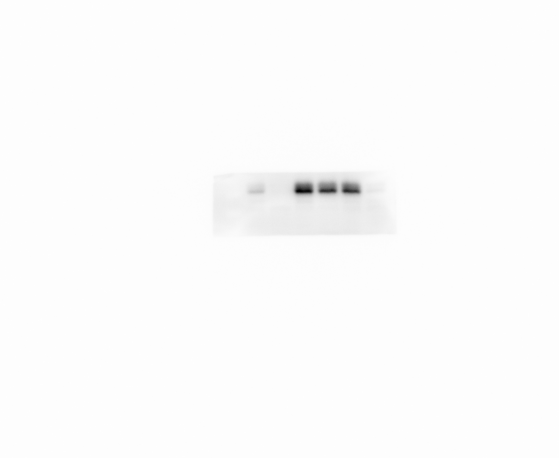

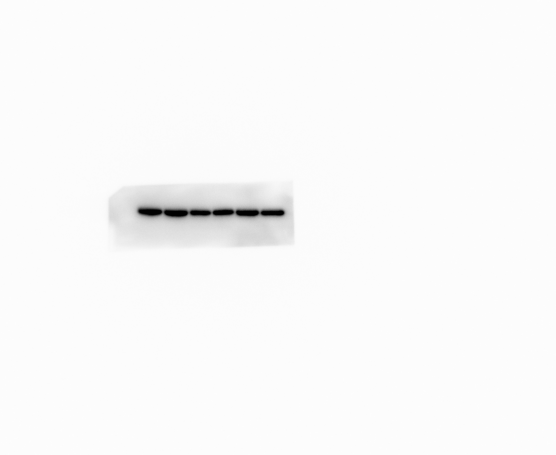


Figure2A:


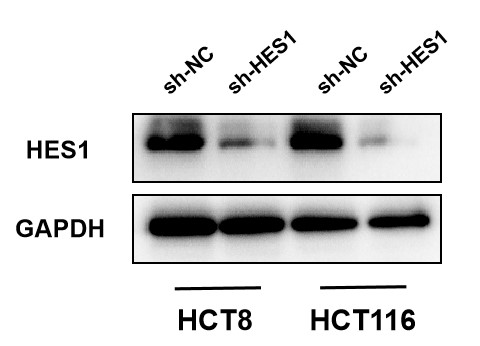


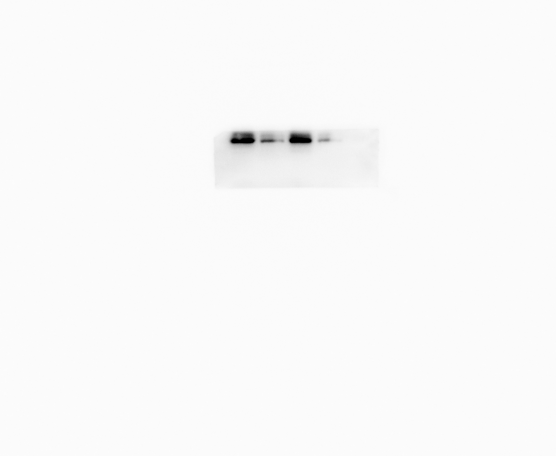

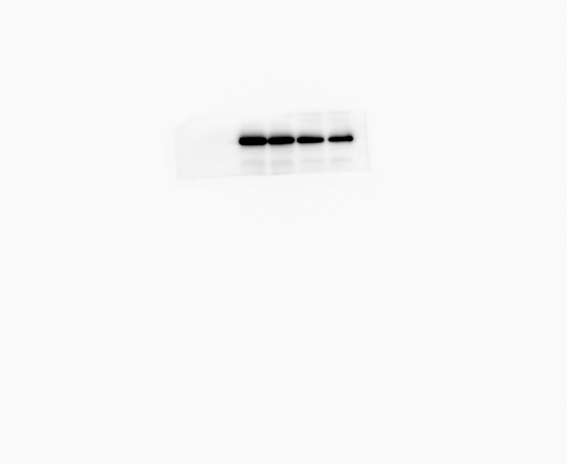


Figure3C:


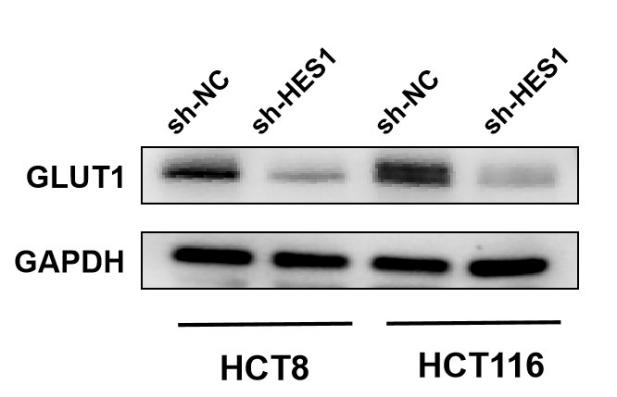


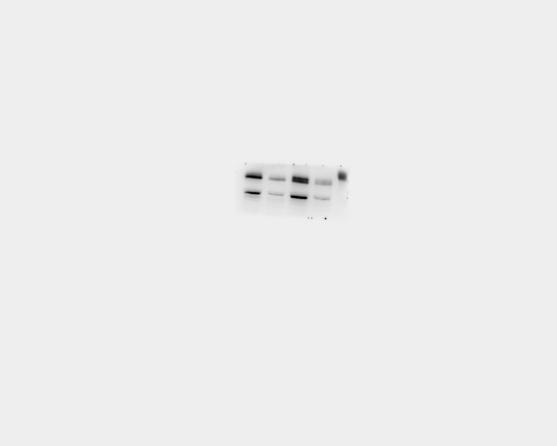

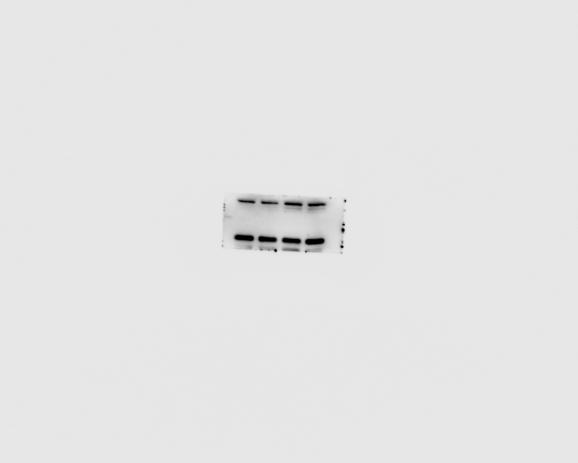


Figure4A:


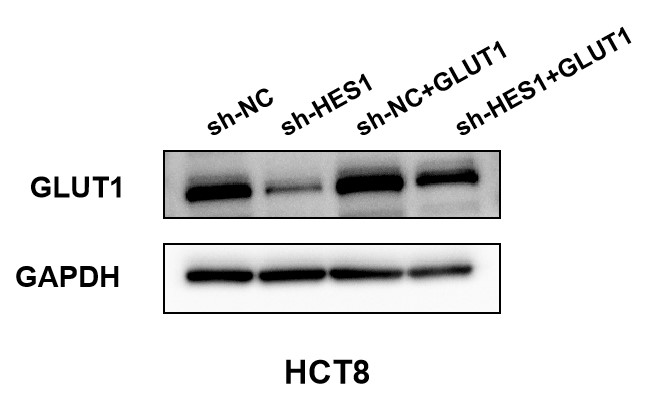


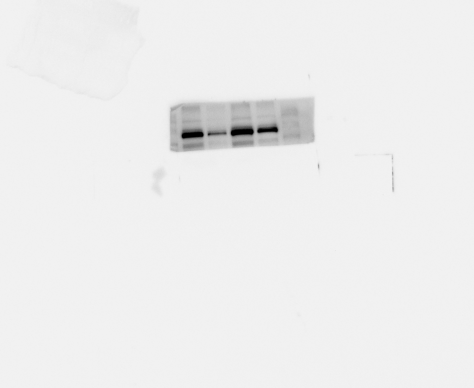

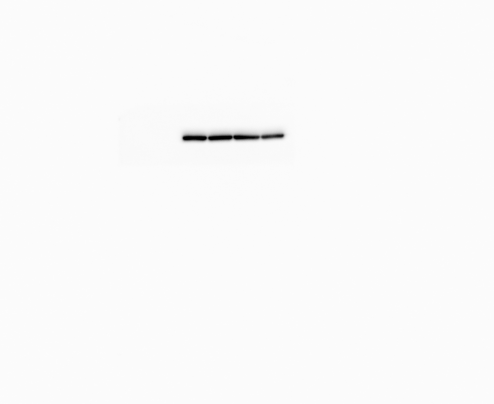

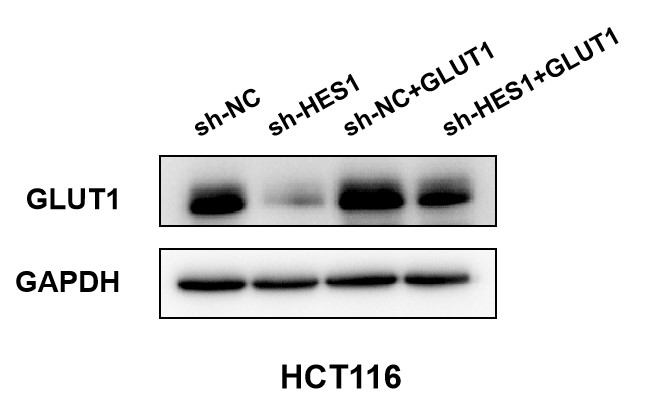


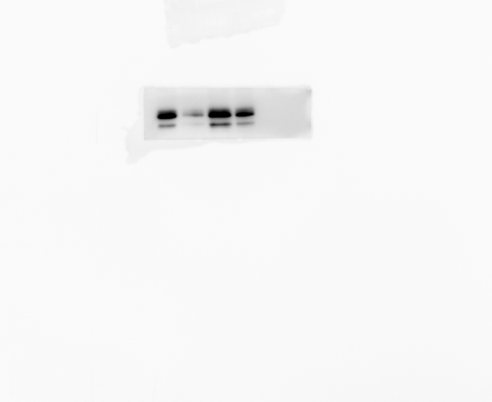

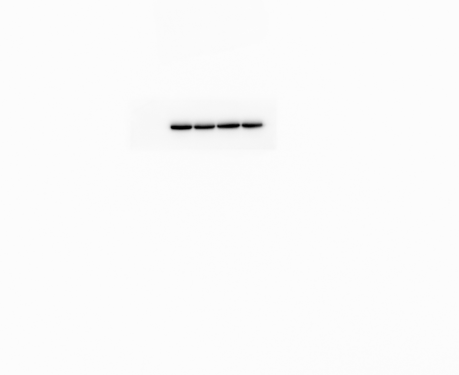


Figure5D:


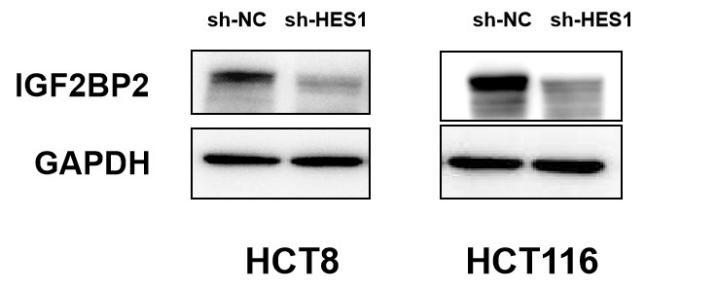


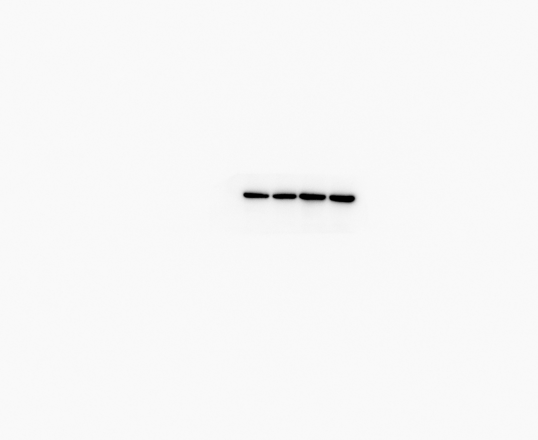

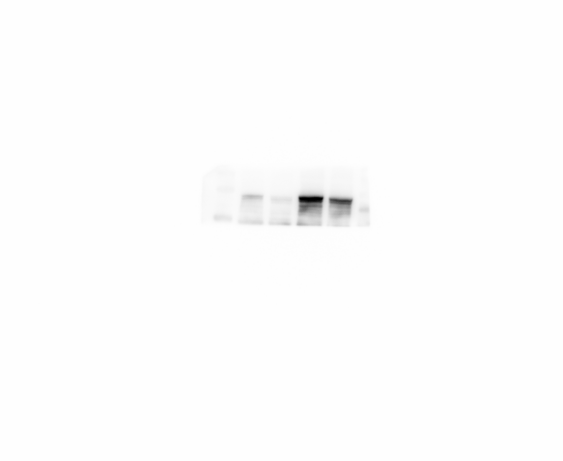

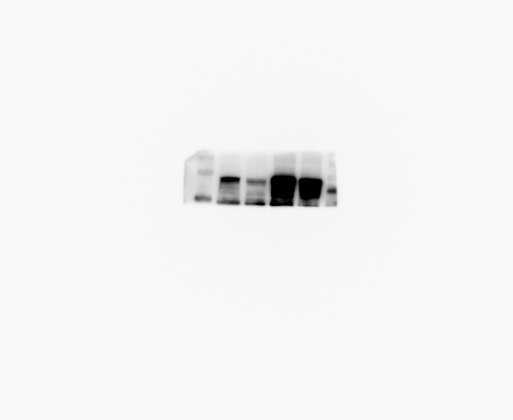


Figure6I:


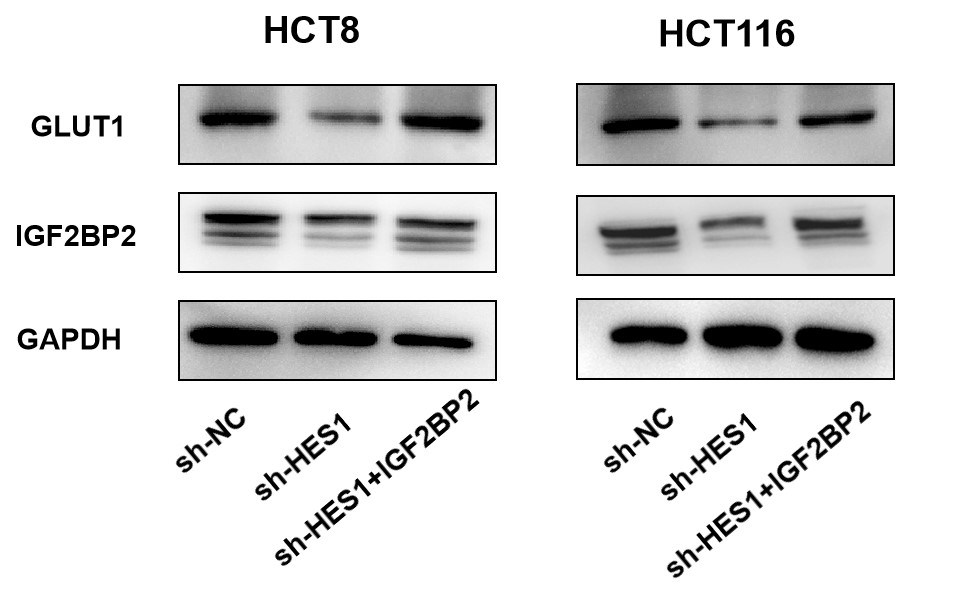


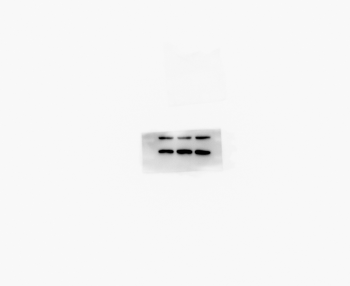

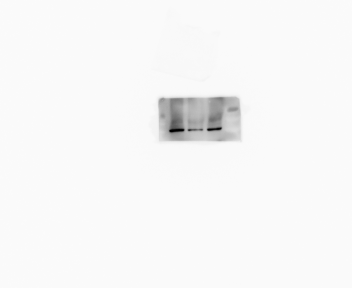

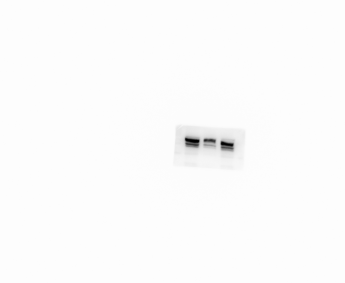


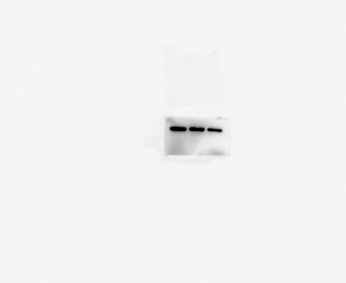

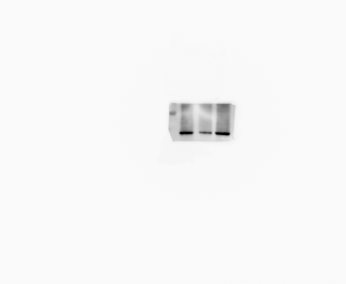

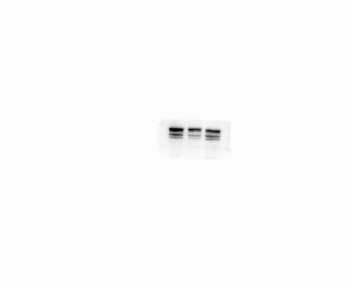

Supplement: Supplementary file 3 — Original Data File [file 41420_2023_1707_MOESM3_ESM.docx]
